# Does the need for uniqueness lead to non-suicidal self-injury? The mediation of depression and the moderation of gender

**DOI:** 10.3389/fpubh.2023.1198483

**Published:** 2023-09-12

**Authors:** Xian Zhang, Wanjun Cao, Jiashuai Fang, Dongxu Hu

**Affiliations:** ^1^Student Counselling and Mental Health Center, Qingdao University, Qingdao, China; ^2^Normal College, Qingdao University, Qingdao, China; ^3^Faculty of Education, Central China Normal University, Wuhan, China; ^4^College of Computer Science and Technology, Qingdao University, Qingdao, China

**Keywords:** demoralization, depression, gender role, interpersonal relations, need for uniqueness, non-suicidal self-injury, suicide, suicide attempt

## Abstract

**Objective:**

Based on the integrated theoretical model of the development and maintenance of non-suicidal self-injury (NSSI) and gender role theory, this study investigated the longitudinal impact of the need for uniqueness on NSSI among adolescents, and the mediating role of depression and the moderating role of gender.

**Participants:**

A total of 1,166 middle school students (*Mean _age_* = 13.04, *SD_age_* = 0.78, range = 11–16) from a city in central China was recruited to complete the Need for Uniqueness Scale, Depression Scale, and Adolescent Self-Injury Questionnaire at two waves. The participants included 475 boys and 457 girls.

**Methods:**

Convenience sampling was used, and a longitudinal study (2 time points with a 6-month interval) was conducted to test our hypotheses. SPSS 25.0 was used to evaluate reliability, and to calculate descriptive statistics and Pearson correlation. PROCESS version 3 was used to test longitudinal relationships among the need for uniqueness, depression and NSSI, and construct a moderated mediation model.

**Results:**

Results revealed that T1 need for uniqueness in adolescents was significantly positively associated with T2 NSSI and T2 depression, and T2 depression was significantly positively associated with T2 NSSI. After controlling for gender, T1 need for uniqueness positively predicted T2 NSSI. Furthermore, the mediation analysis demonstrated that the pathway linking T1 need for uniqueness to T2 NSSI through T2 depression was statistically significant. Moreover, gender moderated the indirect effect from T2 depression to T2 NSSI in the association between T1 need for uniqueness and T2 NSSI. Compared to boys in the same situation, girls who are susceptible to depression were more likely to commit NSSI.

**Conclusion:**

This study demonstrated that the need for uniqueness in adolescents longitudinally predicts NSSI through the mediating role of depression and gender moderates the indirect effect from depression to NSSI. The current study not only suggests that the need for uniqueness is a risk factor for NSSI among adolescents, but also provides an empirical basis for the prevention and intervention of NSSI.

## 1. Introduction

Self-injury has become a significant public health problem among adolescents worldwide (1, 2). Non-suicidal self-injury (NSSI) refers to individuals intentionally harming themselves without the intention of suicide, including behaviors such as cutting themselves with a knife, burning themselves with cigarettes, intentionally hitting themselves, and other deliberate, direct damage or altering of bodily tissue behaviors (3, 4). The prevalence rate of NSSI among adolescents can be up to 57% (5). Specifically, a meta-analysis reported an aggregate lifetime prevalence of NSSI in Chinese adolescents at 22.1%, with a 12-month prevalence of 19.5% (6), and the prevalence of NSSI in Iranian young adults and adolescents was 4.3 to 40.5% (7). Although an individual suffering from NSSI has no initial intent to commit suicide, previous researchers have found that early NSSI is an important predictor of future suicidal ideation and behavior (8–11). For instance, a meta-analysis of relevant studies conducted in both English and Chinese populations revealed a co-occurrence rate of NSSI and suicide attempt at 26% (12). Furthermore, NSSI is positively associated with many mental disorders, such as borderline personality disorder and depression (13). Given the potential harm of NSSI and adolescents being a high-risk group of NSSI (14, 15), it is noteworthy to explore the factors and underlying mechanisms of NSSI.

Previous studies have showed that the main factors leading to NSSI involve genetic, physical (e.g., biological pain, endocrine system and weakened immune systems) and social facets (e.g., poor socioeconomic status, toxic interpersonal exposures and migration), as well as psychiatric disorders (e.g., Borderline Personality Disorder and sleep disorders) (3, 16–20). Among these factors, the effect of basic psychological needs (autonomy, competence, and relatedness) on NSSI has received some attention from a few researchers (16). However, the influence of the need for uniqueness on NSSI has remained unexplored.

Snyder and Fromkin (21) defined the need for uniqueness as the individually psychological need in pursuit of uniqueness and distinctness from other people. In addition, the motivated identity construction theory maintains that the need for uniqueness is theorized as an identity motive, which plays a leading role in the process of identity construction (22). Adolescence is a crucial period of identity construction and adolescents are prone to identity crisis (23). Therefore, adolescents may seek to fulfill the need for uniqueness as a way to deal with identity crisis. However, the need for uniqueness may lead to social punishment particularly within collectivist culture where conformity and group harmony are highly regarded (24).

Previous studies have indicated that the need for uniqueness is positively associated with negative emotions, such as anxiety and depression (25). As negative emotions are often a significant trigger of NSSI in adolescents (26), we assume that the need for uniqueness may be positively associated with NSSI in adolescents. Although China is considered a typical collectivist society, people’s need for uniqueness has dramatically risen in recent years, likely influenced by Western individualistic culture (27). In sum, investigating the relationship between the need for uniqueness and NSSI among adolescents holds both theoretical and practical implications.

### 1.1. Need for uniqueness and NSSI

The need for uniqueness is a universal psychological need emphasizing individual desire for being different from others. Driven by this psychological need, people will display their uniqueness through various behaviors. For example, when choosing names and nicknames, individuals with a higher need for uniqueness tend to choose more uncommon names than do their counterparts (27, 28). With respect to shopping, individuals high in need for uniqueness prefer unconventional product appearances and niche brands and often avoid the popular choices (29).

Although displaying uniqueness can satisfy Chinese adolescents’ psychological needs, it may trigger social ostracism or rejection (30, 31). The pursuit of uniqueness is widely encouraged within individualistic culture, whereas, unwelcome within collectivistic culture (32). Individuals who display uniqueness in China, generally considered a collectivistic country, may be seen as social misfits who challenge collective consistency and violate social norms (33). Therefore, individuals who manifest uniqueness may face social punishment such as social ostracism and rejection from other members within the collective culture (34).

According to the social signaling hypothesis, NSSI can be an effective strategy to communicate social signals in certain situations despite the physical pain it brings (3, 35). When adolescents encounter bullying or difficult situations, NSSI may serve as a powerful way to convey their pain, despair or anger to other people (36). For instance, social ostracism or rejection can be particularly distressing for adolescents because they tend to prioritize peer relationships (37, 38). As adolescents often lack the social skills to manage such situations through normal communication, they may deliver how deeply they have been hurt *via* NSSI to the people who, they suppose, may care about them (25, 39). Previous studies have shown that adolescents who experience peer ostracism, rejection, or bullying have a higher risk of self-harm (15, 40). Thus, adolescents who feel isolated or rejected due to manifestation of uniqueness are likely to resort to NSSI to express their difficult experiences (41).

Hereby, we propose Hypothesis,

*H1*: The need for uniqueness is positively associated with NSSI.

### 1.2. The mediating role of depression

To intervene in NSSI more effectively, it is also necessary to study the underlying mechanism on the path that the need for uniqueness leads to NSSI. According to the integrated theoretical model of the development and maintenance of NSSI, NSSI can be influenced by both distal risk factors (e.g., childhood abuse, familial hostility) and intrapersonal vulnerability factors (e.g., high aversive emotions), and that distal risk factors function through intrapersonal vulnerability factors (42). In this model, the need for uniqueness is considered as a distal risk factor, while depression is considered as an intrapersonal vulnerability factor (42). The model also indicates that the distal risk factor of the need for uniqueness may influence NSSI through the intrapersonal vulnerability factor of depression (43–46).

On the one hand, the need for uniqueness may be positively associated with depression. Depression is a typical negative emotion characterized by low mood, loss of interest, and a sense of worthlessness (47, 48). Beck’s cognitive theory of depression proposes that negative cognition about the world and the self-play a critical role in the development of depression (49). The need for uniqueness may lead to negative cognition about the world in which they live. China is a typical collectivistic culture where the pursuit of uniqueness is not encouraged (25, 32). This social environment can be challenging for adolescents high in need for uniqueness, who may feel that their needs cannot be met and their true selves cannot be expressed (50) leading to the dissatisfaction with their circumstances and a negative cognition about the world, for example, “I live in a very bad world.”

Furthermore, the need for uniqueness may lead to a negative cognition about the self. According to the looking-glass self-theory, people often form their self-perception by referring to social feedback (51). Within collectivist culture, unique behaviors often go against the conventional consensus and disrupt collective consistency, leading to individual dissatisfaction about the group and rejection from other group members (34). Prolonged rejection by group members may cause Chinese adolescents to develop negative self-images (50, 52). For instance, those high in need for uniqueness outcasts may feel frustrated in this situation and even may view themselves as undesirable figures in social circumstances (53).

On the other hand, depression may be positively associated with NSSI among adolescents. The experience avoidance model posits that when individuals experience negative emotions which are difficult to endure and regulate, they may resort to NSSI as an extreme means of escape (26, 54). Depression is a painfully negative emotion, and adolescents usually lack adequate emotional regulation skills to cope with it (55, 56). When depression strikes, adolescents may feel as if they are sinking into a swamp and instinctively seek to escape (57). Nevertheless, NSSI is often accompanied by intensely physical pain, which acts as a ways of attention shifting to help adolescents escape from negative emotions. The acutely physical pain can readily capture an individual’s attention and shift their focus away from internal emotional experiences to external physical experiences (26, 54, 58). Suppressing the psychological pain of depression by bearing the physical pain of NSSI, adolescents may consider NSSI to be an effective means to avoid negative emotions (59, 60). Empirical studies also revealed a positive correlation between adolescent depression and NSSI (40, 61).

Thus, we propose Hypothesis,

*H2*: Depression mediates the association between the need for uniqueness and NSSI; the need for uniqueness is positively associated with depression, which is positively associated with NSSI.

### 1.3. The moderating role of gender

Drawing from the experiential avoidance model, although negative emotions such as depression and anxiety can trigger NSSI, this process is influenced by the individual’s ability to regulate negative emotions (26). When individuals are incapable of regulating their negative emotions which are caused by stressful events, NSSI may become the priority option to relieve the intensely painful feelings (62–64). Previous studies have found that the ability and styles of regulating negative emotions differ significantly between genders (62–66). Therefore, gender may moderate the relationship between negative emotions (e.g., depression) and NSSI. More precisely, female adolescents experiencing depression are at a greater likelihood of engaging in NSSI than their male counterparts experiencing depression.

According to gender role theory, men and women are given different social expectations during socialization and their social behaviors are guided by different social norms, resulting in different ways of thinking, feeling, and behaving (67, 68). Men are commonly expected to take on the roles which emphasize responsibility, patience, and emotional stability (63). Those expectations require men to exercise strong emotional control and avoid excessive emotional involvement (65, 69). In contrast, women are often assigned gender roles where society tends to tolerate their emotional expression, even allowing them to behave petulantly (67, 68). These distinct gender role expectations lead to differences in the development of emotional regulation skills and styles. Men usually have stronger emotional regulation efficacy and more dispassionate emotional regulation styles than do women (62–66). Empirical studies have shown that females are more likely to ruminate when experiencing sadness or depression, compared to males (65, 70, 71). For men with depressive symptoms, their stronger sense of emotional regulation efficacy and more dispassionate emotional regulation styles (e.g., cognitive reappraisal) (62, 63, 65, 66), may protect them from turning to NSSI for emotional regulation. However, women with depressive symptoms may have weaker emotional regulation efficacy and more distressing emotional regulation styles, for example, rumination (65, 70, 71). Therefore, they may be more likely to regulate their emotions by NSSI than men.

**Figure 1:**
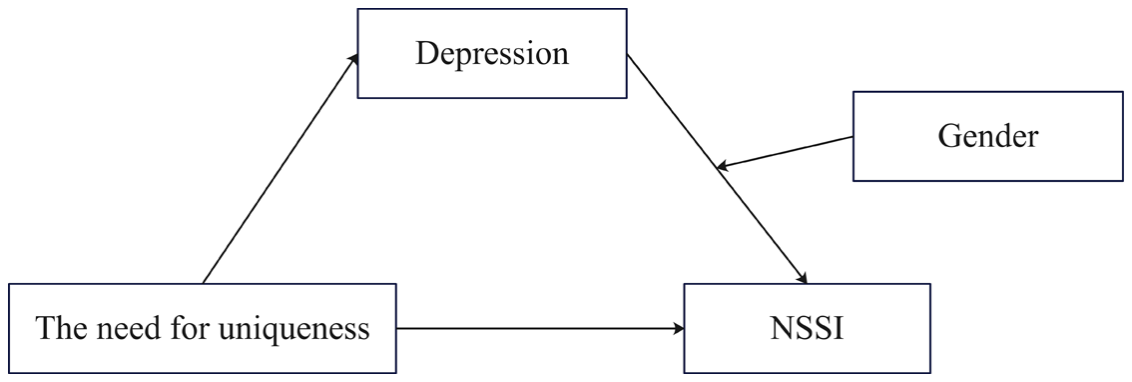
The proposed moderated mediation model.

Accordingly, we propose Hypothesis H3: Gender moderates the indirect effect of depression on NSSI in the association between the need for uniqueness and NSSI (Figure 1). Compared to girls, depression has a greater impact on among boys.

## 2. Methods

### 2.1. Participants and design

The present study received approval from the Ethics Committee for Scientific Research at Qingdao University, indicating that the study design and methods adhere to established ethical standards in scientific research. The sampling method utilized in this study was convenience sampling. Graduate students specializing in psychology and trained in research methodology carried out this investigation. A total of 1,166 adolescents were recruited from three middle schools located in a city in central China. Prior to formal testing, consent was obtained from participants and their parents or guardians for the use of their responses in our research. We ensured that they fully understood each step of the survey process through appropriate ways such as providing detailed instructions and sufficient time for questions, assisting participants with difficult questions, and emphasizing that participation was optional and anonymous.

The study involved two assessments with a 6-month interval (Time 1 and Time 2). T1 collecting demographic variables as controlling variables and the need for uniqueness as an independent variable. T2 measured depression as a mediating variable and NSSI as a dependent variable.

The survey includes 1,166 adolescent participants at Time 1. Among them, 541 were females, and 601 were males. Six months later in Time 2, the students’ participation number decreased from 1,166 to 970. Attrition (196 students) resulted from participants’ absenteeism or refusal to participate in the study. Additionally, 38 students’ answers were excluded due to incompletion. Consequently, the final sample for analysis comprised 932 middle school students, which comprised 457 (49.03%) females and 475 (50.97%) males. Their ages range from 11 to 16 (*M* = 13.04, *SD* = 0.78).

### 2.2. Measures

#### 2.2.1. Need for uniqueness

Need for Uniqueness Scale developed by Lynn and Harris (69) was used in the study. The scale was revised into a Chinese version by Cai and Zou (27) with good reliability and validity. It consists of 4 items, such as “It’s very important for me to be different.” A total of 4 items were scored on a five-point Likert scale with 1 (strongly disagree) to 5 (strongly agree). Higher scores indicated a greater need for uniqueness among the adolescents. The Cronbach’s alpha coefficient for the scale was calculated to be 0.86.

#### 2.2.2. Depression

In this study, we utilized the Depression-Anxiety-Stress Scale-21 (DASS-21) developed by (72), which has a revised Chinese version (45). To measure depression, we used the depression subscale, which includes 7 items, such as “I find it difficult to take the initiative to start working,” and responses were scored on a five-point Likert scale ranging from “0” for “not at all” to “3” for “very much.” Higher scores indicated a higher level of depression. The Cronbach’s alpha coefficient for the scale used in this study was calculated to be 0.86.

#### 2.2.3. Non-suicidal self-injury

The Adolescent Self-Injury Questionnaire was revised into a Chinese version by Feng (43) with good reliability and validity. The questionnaire consists of 18 items, such as “Using a cigarette, lighter, or other instruments, deliberately burn or scald your skin.” The frequency of NSSI was examined on four levels: 0 times, 1 time, 2–4 times, and more than 5 times (including 5 times). The mean scores across the 18 items were used to assess NSSI. Higher scores indicate more instances of NSSI for the adolescents. Cronbach’s alpha for the scale in this study was 0.90.

### 2.3. Data analysis

In this study, all data analyses were performed using SPSS 25.0. First, descriptive statistics and Pearson correlation analysis were conducted to explore the data. Second, PROCESS version 3 was used to test longitudinal relationships among the need for uniqueness, depression and NSSI, and construct a moderated mediation model (73). After standardizing all continuous variables, gender was converted into a dummy variable, with boys coded as 0 and girls coded as 1. For the purpose of approximating the confidence interval (CI) of the indirect impact, 5,000 boot-strapped samples were created. Statistical significance was demonstrated by a 95% bias-corrected accelerated CI that did not contain zero.

## 3. Results

### 3.1. Preliminary analyses

Means, standard deviations, and Pearson’s correlations of all variables were calculated and are shown in Table 1. As the results indicated, T1 need for uniqueness was positively associated with T2 depression (*r* = 0.18, *p* < 0.01) and T2 NSSI (*r* = 0.11, *p* < 0.01). T2 depression was positively associated with T2 NSSI (*r* = 0.51, *p* < 0.01).

**Table 1:** Means, standard deviations, and correlations for the main variables (*N* = 932).

Variables	*M*	*SD*	1	2	3
1. T1 Need for uniqueness	2.05	0.99	–		
2. T2 Depression	0.59	0.71	0.18^**^	–	
3. T2 NSSI	1.22	0.38	0.11^**^	0.51^**^	–

*N* = 932. **p < 0.01. NSSI, non-suicidal self-injury.

### 3.2. Mediation analyses

After all variables were standardized and gender was converted into a dummy variable, with boys coded as 0 and girls coded as 1, Model 4 of PROCESS (73) was conducted to examine the longitudinal impact of need for uniqueness on NSSI among adolescents as well as the possible mediating effect of depression. As indicated in Table 2, after controlling for gender, T1 need for uniqueness positively predicted T2 NSSI (*β* = 0.10, *p* < 0.01, Eq.1); T1 need for uniqueness positively predicted T2 depression (*β* = 0.18, *p* < 0.001, Eq. 2), the direct association between T1 need for uniqueness and T2 NSSI was not significant (*β* = 0.01, *p* > 0.05), and T2 depression positively predicted T2 NSSI (*β* = 0.50, *p* < 0.001, Eq.3). The bias-corrected bootstrapping mediation test illustrated that the process by which T1 need for uniqueness predicted T2 NSSI through T2 depression was significant, *indirect effect* = 0.09, *Boot SE* = 0.02, 95% *CI* = [0.06,0.13]. The results of the mediation analysis support H1 and H2.

**Table 2:** The mediation model.

Predictors	Equation 1 (Criterion = T2 NSSI)			Equation 2 (Criterion = T2 Depression)			Equation 3 (Criterion = T2 NSSI)		
	*β*	*SE*	*t*	*β*	*SE*	*t*	*β*	*SE*	*t*
Gender	0.10	0.07	1.59	0.10	0.06	1.55	0.05	0.06	0.94
T1 Need for uniqueness	0.10	0.03	3.20^**^	0.18	0.03	5.61^***^	0.01	0.03	0.46
T2 Depression							0.50	0.03	17.47^***^
*R^2^*	0.01			0.04			0.26		
*F*	6.43^**^			17.02^***^			107.43^***^		

**p < 0.01, ***p < 0.001. NSSI, non-suicidal self-injury.

### 3.3. Moderation analyses

**Figure 2:**
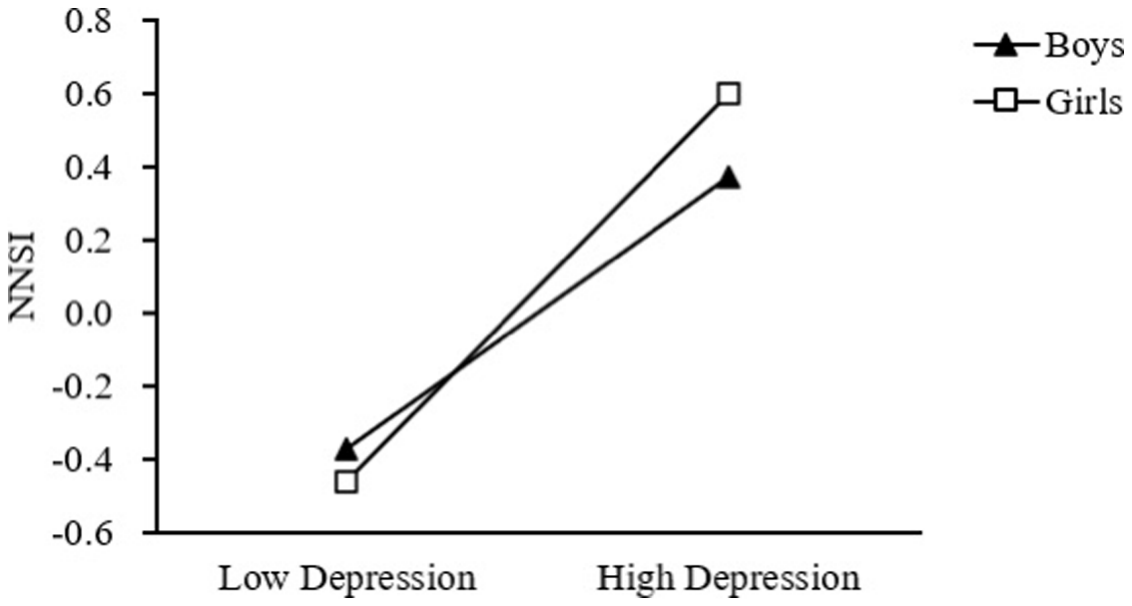
Gender moderated the relationship between depression and NSSI.

Model 14 of PROCESS (73) was conducted to investigate whether gender moderated the association between T2depression and T2 NSSI. The results of the moderation analyses were showed in Table 3. The results of the moderation analysis support H3. The regression model suggested the T1 need for uniqueness was not a significant predictor of T2 NSSI (*β* = 0.01, *t* = 0.47, *p* > 0. 05), while T2 depression positively predicted T2 NSSI (*β* = 0.42, *t* = 10.38, *p* < 0.001). Moreover, the interaction between gender and T2 depression was associated with T2 NSSI (*β* = 0.17, *t* = 3.01, *p* < 0.01), with a 95% confidence interval of [0.06, 0.28] and did not contain 0. The results indicated that gender played a moderating role in the relationship between T2 depression and T2 NSSI, and hypothesis H3 was tested. As illustrated in Figure 2, simple slope tests indicated that compared with boys (*β* = 0.42, *t* = 10.38, *p* < 0.05), T2 depression has a greater impact on T2 NSSI in girls (*β* = 0.59, *t* = 14.64, *p* < 0.01).

**Table 3:** The moderation model.

Predictors	Equation (Criterion = T2 NSSI)		
	*β*	*SE*	*t*
Gender	0.05	0.06	0.95
T1 Need for uniqueness	0.01	0.03	0.47
T2 Depression	0.42	0.04	10.38^***^
T2 Depression × Gender	0.17	0.06	3.01^**^
*R^2^*	0.27		
*F*	83.54***		

**p < 0.01, ***p < 0.001. NSSI, non-suicidal self-injury.

## 4. Discussion

Drawing on the integrated theoretical model of the development and maintenance of NSSI, we investigated the longitudinal impact of T1 need for uniqueness on T2 NSSI among adolescents in China. Additionally, we examined the mediation of depression and the moderation of gender. Results revealed that T1 need for uniqueness in adolescents was significantly positively associated with T2 NSSI and T2 depression, and T2 depression was significantly positively associated with T2 NSSI. After controlling for gender, T1 need for uniqueness positively predicted T2 NSSI. Furthermore, the mediation analysis demonstrated that the pathway linking T1 need for uniqueness to T2 NSSI through T2 depression was statistically significant. Moreover, gender moderated the indirect effect from T2 depression to T2 NSSI in the association between T1 need for uniqueness and T2 NSSI. Compared to boys in the same situation, girls who are susceptible to depression were more likely to commit NSSI.

### 4.1. Relationship between the need for uniqueness and NSSI among adolescents

This study indicated that the T1 need for uniqueness was positively associated with T2 NSSI among adolescents, which supported hypothesis H1. The results surprisingly revealed an adverse effect of the need for uniqueness on adolescents’ mental and behavioral problems (53, 74), and broadened the research area of the need for uniqueness. Previous research suggested that NSSI was associated with basic psychological need for competence, autonomy, and relatedness (16, 75), but few researchers focused on the need for uniqueness. According to the optimal distinctiveness theory, the need for uniqueness is closely related to individual adaptation (30, 76).

Since China has undergone significant cultural and psychological changes as a result of social and economic transformation during the past century, the contemporary Chinese society has witnessed the rise of individualistic values and the collapse of traditional values (77, 78). Although the need for uniqueness among adolescents is increasing in China (27), previous research studies focused mainly on the field of marketing (29, 79) and few studies have examined the need for uniqueness from the perspective of adolescent development. This study found that adolescents high in need for uniqueness were more likely to engage in NSSI, indicating that the need for uniqueness is a risk factor for adolescents’ NSSI.

Therefore, we suggest that the family, educational system and the public be aware of the potential danger of adolescents’ pursuit of uniqueness. It might be considered universally effective for adolescents across the world too, satisfy the need for uniqueness by wearing unusual clothes or cutting maverick hair (80). Within collectivist culture, however, doing so may violate the values and norms of collectivism and possibly leads to social ostracism and peer bullying (34). Based on the social signaling hypothesis, adolescents who experience bullying and social exclusion may vent their pain by harming themselves, considering that they usually lack mature social skills to handle these issues (3, 35). Recent researches have also revealed that adolescents’ craving for uniqueness causes negative outcomes, such as anxiety and addictive behaviors (81).

### 4.2. The mediating role of depression

The present study demonstrated that the T1 need for uniqueness among adolescents was positively associated with T2 NSSI through the mediating role of T2 depression, which was consistent with hypothesis H2. According to the person-environment fit theory, how well adolescents adapt to their environment depends on how well their individual characteristics match that environment (82). Chinese adolescents with higher need for uniqueness often desire to be different and stand out from the crowd. Nonetheless, their need for uniqueness is incompatible with the demands of the collectivist environment, which may hinder personal growth and lead to maladjustments, for instance, depression and NSSI (81).

The results of this study supported the integrated theoretical model of the development and maintenance of NSSI, which maintains that the likelihood of NSSI can result from distal variables through intrapersonal vulnerabilities (3, 35). The study revealed that the need for uniqueness, as a distal risk factor can cause NSSI through intrapersonal vulnerability factor (depression). This result is consistent with previous studies, indicating that individual characteristics, such as self-criticism, alexithymia, distress tolerance, and behavioral inhibition system/behavioral approach systems, influence NSSI through the mediating role of negative emotions, such as depression and psychache (46, 83, 84).

The current study not only elucidates a mechanism underlying the association between the need for uniqueness and NSSI, but it also explored and tested another detrimental impact of fulfilling the need for uniqueness, i.e., depression. Furthermore, this study expands the application scope of the integrated theoretical model of the development and maintenance of NSSI.

### 4.3. The moderating role of gender

This present study found that gender moderated the association between T2 depression and T2 NSSI. Specifically, depression had a greater impact on NSSI in girls than in boys, which was consistent with hypothesis H3. Social norms demands more responsibility, patience and emotional stability from men than from women (85). This difference in gender roles results in different regulatory emotional self-efficacies and emotion regulation strategies between men and women when dealing with problems. Specifically, compared to women, men may develop a higher level of regulatory emotional self-efficacy (66) and more composed ways of regulating emotions (62, 63, 65). Previous research has shown that males tend to utilizing cognitive reappraisal strategies when in depressive mood, whereas females are more prone to rumination (71, 86). Based on the experiential avoidance model of NNSI, when girls are incapable of regulating their emotions, they are more likely to escape negative emotions through NSSI that accompanies intense physical pain (26, 54).

An alternative explanation for gender difference is stress response. Adolescents with depressive symptoms may experience low spirits, retarded thinking and decreased motivation (57). Their worsened mental states may suspend the functioning of their study and daily life, which results in a situation where they are under much stress (87, 88). Compared with men, women are more likely to report physical symptoms associated with stress (89). Similarly, it is reasonable to assume that girls who suffer from depressive mood have more physical symptoms than do their boys counterparts. Given that physical symptoms are closely associated with NSSI (20), girls who suffer from depressive mood are more likely than boys in the same situation to conduct NSSI.

The present result was consistent with earlier research, which has found that depression has a greater effect on suicidal thoughts among adolescent girls than among adolescent boys (44, 90). These findings suggest that more attention should be paid to adolescent girls with depression, because they are more vulnerable to NSSI than adolescent boys with depression.

### 4.4. Theoretical and practical implications

An important theoretical implication of our study is that this study is the first to examine the need for uniqueness on NSSI on an adolescent sample. we have found that the need for uniqueness increases NSSI, indicating that a positive individual trait from Western culture may lead to NSSI. This finding deepened our understanding of the factors that contribute to NSSI. Previous research has focused on the effect of explicitly negative characteristics (e.g., self-criticism, low self-esteem, and experiential avoidance) on NSSI (3, 20). Neutral factors or even positive factors in Western culture are relatively neglected.

Our current study centered on one positive individuals characteristic, distinctiveness, which not only is widely acclaimed in western society but epitomizes western values and liberal spirit (24). Uniqueness is a social value widely recognized in mainstream Western culture (30, 32, 91). It is also considered to be a positive individual characteristic and is essential in constructing one’s self-identity and boosting self-esteem (30, 32). Empirical evidence has shown that a sense of distinctiveness is positively associated with optimism, hope, resilience, and positive self-evaluation, and it helps to enhance the individual psychological state (92, 93). Despite its widely accepted advantages, our results disclosed that the need for uniqueness may increase the risk of NSSI.

Another theoretical implication of our study is that this study enriches the theory of person-environment fit (82). According to this theory, individual adaptiveness depends on how well individual characteristics accords with the environment (82, 94). For example, individuals experience higher self-esteem when their personality matches the personality of the city they reside in than a mismatch (94). Similarly, better job performance is observed when individuals correspond to organizations (95). In terms of the current study, individuals adapt to the environment more easily when their need for uniqueness aligns with the cultural environment than when it does not fit the environment. Researchers have also found that while the need for uniqueness increases life satisfaction among American participants, it decreases life satisfaction among Japanese participants (76). Similar in the current study, the need for uniqueness, which is generally known to have positive effects in Western culture, can inflict harm on Chinese adolescents. This finding provides new evidence for the theory of person-environment fit.

Practical implications could also be drawn from the results of this study. Firstly, it is often clinically complex to distinguish between the presence or absence of suicidal intent, direct intervention to lessen suicidal ideation is challenging. However, compared to suicidal ideation, NSSI leaves physical traces on the body, such as cuts and cigarette burns, which implies that NSSI is easier to be discerned than is suicidal ideation (12). Previous studies have demonstrated that NSSI can significantly predict suicidal ideation (96–98). Therefore, to improve the precision of discerning suicidal intent, clinical practitioners can pay specific attention to NSSI in adolescents.

Secondly, as the need for uniqueness in China has been increasing, there is a danger that some adolescents pursue uniqueness through demonstrations of NSSI. Therefore, it is essential for schools and teachers to be aware of the negative impact of the need for uniqueness on NSSI among adolescents. If guiding adolescents to fulfill their need for uniqueness in an healthy manner, the potential negative effects on their mental health might be lessened.

Thirdly, given that practitioners usually utilize emotion regulation therapy to prevent adolescents from engaging in NSSI (99), this finding suggests that such therapy could also be used to assist adolescents who have already conducted NSSI. The government and schools are encouraged to organize psychological consulting or group therapy sessions to facilitate adolescents’ mental health. For example, the negative effects of depression can be alleviated by developing positive emotion regulation strategies and good coping styles in adolescents through diverse approaches, such as group counseling, individual counseling, and public programs, thus reducing the risk of NSSI. Additionally, concerning that eliminating depression or depressive symptoms in a short-term is impractical, therapies such as Acceptance and Commitment Therapy (ACT) can be used to enhance the acceptance of depressive mental state (100, 101), which may further reduce the impact of depression on NSSI.

Finally, gender roles are crucial in dealing with negative emotions, and traditional female roles are not conducive to regulating negative emotions (63). Therefore, the government and schools can encourage parents to refresh their views of gender roles. Especially parents of adolescent girls can provide unbiased advice during gender socialization and guide them to manage negative emotions. Moreover, compared to boys, girls suffer from depression are more likely to conduct NSSI. The government and schools need to provide mental health courses and training related to emotional regulation for girls to improve their emotional regulation strategies and coping skills when experiencing negative emotions such as depression, thereby reducing NSSI.

### 4.5. Limitations

The first limitation was that this study focused on the relationship between the need for uniqueness and NSSI among Chinese adolescents, therefore the results cannot be simply generalized to other countries. According to the person environment fit theory, whether an individual trait is adaptive depends on the degree to which it matches the environment (102, 103). Therefore, the effect of the need for uniqueness on NSSI may vary across different social environments. Previous studies have found that although the need for uniqueness decreases satisfaction with life among Japanese people, it increases satisfaction with life among Americans (76). Therefore, the effect of the need for uniqueness on NSSI in individualistic culture may be potentially different from the present study, which is worth exploring in future cross-cultural studies.

The second limitation was that we only obtained homologous data, which could have led to shared variance among the variables and inflated the significance of the connections that were expected. Future study can recruit participants from various sources, such as parents and peers, to report on the same variables.

The third limitation was that NSSI and depression were not measured at baseline. Previous studies have found that NSSI was affected by both NSSI history and depression (40, 61, 104). However, due to the methodological limitations, we only measured depression and NSSI at Time 2. Future study on the relationship between depression and NSSI should measure NSSI and depression at baseline.

The final limitation is that only one mediator, depression, was considered in the current study. Future studies can test more mediators to further understand the mechanisms underlying the association between the need for uniqueness and NSSI. According to previous literature, we presume that demoralization is a possible mediator. Demoralization features hopelessness or disheartenment, loss of meaning in life, helplessness, sense of failure, and dysphoria (105, 106). Researchers believe that individuals may develop symptoms of demoralization when they cannot meet self and others’ expectations (105, 106). Individuals high in need for uniqueness may find it difficult to live up to the expectations and thus succumb to demoralization. Collectivistic culture underlines consistency and similarity among group members, suggesting that adolescents may be expected by their parents, teachers and peers to behave similar to the way other people do and avoid standing out (33, 34). Therefore, adolescents high in need for uniqueness may be tormented by not meeting others’ social expectations. In addition, displaying uniqueness may not only be disapproved, but even socially punished (24, 31). Under the circumstances, adolescents high in need for uniqueness are not allowed to be the authentic, unique self, i.e., fail to meet their own expectations. Furthermore, we assume that demoralization leads to NSSI. Individuals who suffer from demoralization may be desperate for escapism (106, 107), actions whose goal is to distract the psychological pain by enduring physical pain. In this situation, NSSI as a form of distraction would appeal to those individuals.

## 5. Conclusion

In conclusion, this study enhanced our comprehension of the risk factors that contribute to NSSI among Chinese adolescents, as well as the mechanism that how the need for uniqueness longitudinally leads to NSSI among adolescents in China. Specifically, the results indicate that depression acts as a mediator on the association between the need for uniqueness and NSSI, and that the association between depression and NSSI is moderated by gender. Compared to boys in the same situation, girls who are susceptible to depression were more likely to commit NSSI. The moderated mediation model provides valuable insights into the theoretical exploration of the need for uniqueness and NSSI, and offer potential avenues for relevant intervention.

## Data availability statement

The raw data supporting the conclusions of this article will be made available by the authors, without undue reservation.

## Author contributions

XZ, WC, and JF: conceptualization and methodology. XZ, WC, JF, and DH: writing – original draft. All authors contributed to the article and approved the submitted version.

## Funding

This research was supported by the Qingdao Social Science Planning Project Foundation (Grant number: GDSKL2201064) and the Special Project on Traditional Culture and Economic and Social Development of the Shandong Cultural and Artistic Science Association (Grant number: L2021C10290042).

## Conflict of interest

The authors declare that the research was conducted in the absence of any commercial or financial relationships that could be construed as a potential conflict of interest.

## Publisher’s note

All claims expressed in this article are solely those of the authors and do not necessarily represent those of their affiliated organizations, or those of the publisher, the editors and the reviewers. Any product that may be evaluated in this article, or claim that may be made by its manufacturer, is not guaranteed or endorsed by the publisher.
